# Crystal Engineering of Supramolecular 1,4‐Benzene Bisamides by Side‐Chain Modification – Towards Tuneable Anisotropic Morphologies and Surfaces

**DOI:** 10.1002/cphc.202100597

**Published:** 2021-11-08

**Authors:** Kasper P. van der Zwan, Christoph Steinlein, Klaus Kreger, Hans‐Werner Schmidt, Jürgen Senker

**Affiliations:** ^1^ Inorganic Chemistry III and North Bavarian NMR Center University of Bayreuth Universitätsstraße 30 95447 Bayreuth Germany; ^2^ Macromolecular Chemistry and Bavarian Polymer Institute University of Bayreuth Universitätsstraße 30 95447 Bayreuth Germany

**Keywords:** ab initio structure solution, DFT calculations, NMR crystallography, self-assembly, supramolecular polymer additives

## Abstract

Benzene bisamides are promising building blocks for supramolecular nano‐objects. Their functionality depends on morphology and surface properties. However, a direct link between surface properties and molecular structure itself is missing for this material class. Here, we investigate this interplay for two series of 1,4‐benzene bisamides with symmetric and asymmetric peripheral substitution. We elucidated the crystal structures, determined the nano‐object morphologies and derived the wetting behaviour of the preferentially exposed surfaces. The crystal structures were solved by combining single‐crystal and powder X‐ray diffraction, solid‐state NMR spectroscopy and computational modelling. Bulky side groups, here t‐butyl groups, serve as a structure‐directing motif into a packing pattern, which favours the formation of thin platelets. The use of slim peripheral groups on both sides, in our case linear perfluorinated, alkyl chains, self‐assemble the benzene bisamides into a second packing pattern which leads to ribbon‐like nano‐objects. For both packing types, the preferentially exposed surfaces consist of the ends of the peripheral groups. Asymmetric substitution with bulky and slim groups leads to an ordered alternating arrangement of the groups exposed to the surface. This allows the hydrophobicity of the surfaces to be gradually altered. We thus identified two leitmotifs for molecular packings of benzene bisamides providing the missing link between the molecular structure, the anisotropic morphologies and adjustable surface properties of the supramolecular nano‐objects.

## Graphical abstract

**The crystal structures** of two series of 1,4‐benzene bisamides with symmetric and asymmetric peripheral substitution were solved by NMR crystallography. The authors found that the bulkiness of the side groups guides the supramolecular self‐assembly into one of two specific packing patterns. Their anisotropic crystal‐growth behavior results in a defined nano‐object morphology. Slim side chains, in our case perfluorinated alky chains, led to ribbon‐like nano‐objects, whereas bulky side chains led to thin platelets.

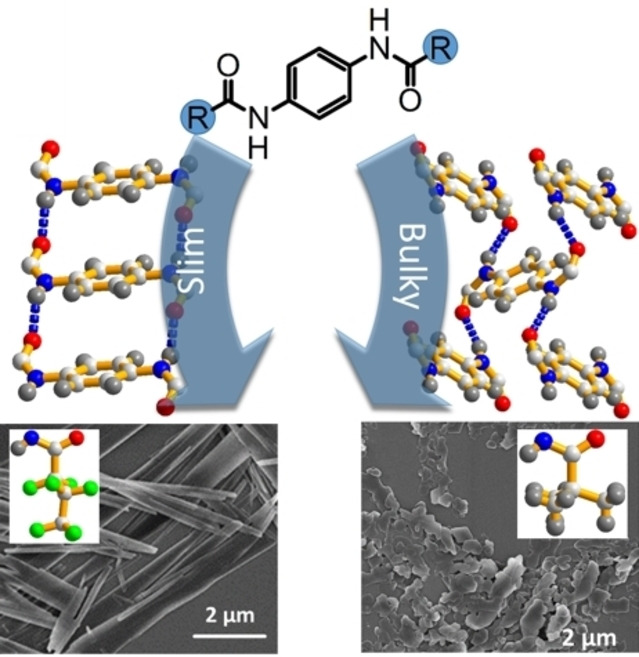

## 1 Introduction

Two‐dimensional nanostructures emerge as an advanced materials class that can be applied in various fields such as sensing, separation and electronics.^[1]^ Among the variety of building blocks for these architectures, hydrogen‐bonded supramolecular systems like bis‐acylurea,^[2]^ cyclic dipeptide^[3]^ and peptoid^[4]^ derivatives remsemble an interesting class of molecules since their high melting points allow for their use as supramolecular polymer additives. These additives rely on *in situ* formed solid‐state nanoobjects in the polymer melt.[^5^, ^6^, ^7^, ^8^, ^9^] Prominent examples are hydrogen‐bonded structures based on sorbitols,[^10^, ^11^] diacids,^[12]^ benzene trisamides[^5^, ^6^, ^7^, ^13^, ^14^] and various bisamides derivatives.[^8^, ^15^] These nanoobjects are formed *in situ* due to the reversible nature of their secondary interactions^[16]^ during processing at elevated temperatures. This results in a high degree of dispersion of the objects within the polymer matrix. When these additives are used as nucleating agents for semi‐crystalline polymers such as *i*‐PP,[^5^, ^8^, ^13^, ^17^] PVDF,^[18]^ and PLA,^[19]^ function and efficiency arises also from the epitaxial match of the nanoobjects’ surface with the polymer crystal.[^10^, ^20^] This in turn allows to control the polymer solid‐state morphology in terms of crystal size and shape as well as crystal modification.

For the design of supramolecular additives, however, a fundamental understanding is required, how the supramolecular structure translates into specific packing patterns and anisotropic crystal morphologies. In particular, two concepts were derived to reach this goal. Crystal engineering^[21]^ provides motifs to predict the formation of specific packing patterns. They should favour the anisotropic growth of the obtained nanoobjects, which in turn can be enhanced by morphology engineering.^[22]^ In this context, crystal engineering relies on anisotropic supramolecular synthons^[23]^ based on a combination of π‐stacking,^[24]^ hydrogen[^25^, ^26^] or halogen^[27]^ bonds that are usually programmed into the supramolecular structure. Examples on how this effects the macroscopic structure are summarized in a recent perspective^[28]^ and a recent review article.^[29]^

Applied to 1,4‐benzene bisamides the two amide groups are the foundation of the supramolecular synthon with the possibility to form two acceptor and two donor hydrogen bonds each. Up to now, two supramolecular synthons have been observed. Either one molecule forms hydrogen bonds to two[^30^, ^31^, ^32^, ^33^] or to four[^34^, ^35^, ^36^, ^37^] other molecules. In the first case a linear arrangement of the molecules arises that features a packing with strong interactions in one dimension.[^30^, ^31^, ^32^, ^33^] This implies the growth of an object with one preferred growth rate and an anisotropic morphology. In the second case, the twisted arrangement of the five molecules involved in the supramolecular synthon induces packings with strong interactions in two dimensions.[^34^, ^35^, ^36^, ^37^]

**Figure 1:**
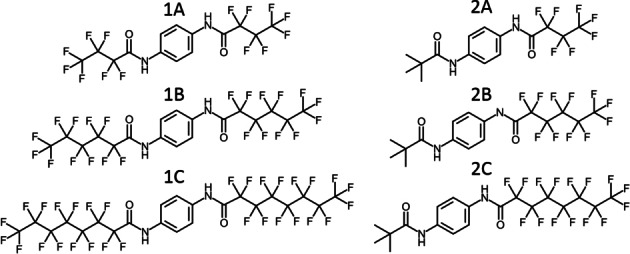
Molecular structures of the target compounds: **1** **A**–**1** **C** symmetrically substituted 1,4‐benzene bisamides and **2** **A**–**2** **C** asymmetrically substituted 1,4‐benzene bisamides.

However, a detailed understanding how the side groups have to be designed to guide into a specific packing pattern and in turn programme a one or two directions of preferred growth is missing. Therefore, we investigate two series of 1,4‐benzene bisamides (Figure 1) featuring a symmetric peripheral substitution with linear perfluorinated alkyl chains (series **1**) and an asymmetric peripheral substitution pattern with a *tert*‐butyl (*t*‐Bu) group and a linear perfluorinated alkyl chain (series **2**). The side chains vary in size and shape but do not introduce additional hydrogen bond acceptors or donors. This allows to probe, whether their steric demand^[38]^ is able to switch between characteristic supramolecular synthons for 1,4‐benzene bisamides. To avoid a potential odd‐even effect, we incremented the chain length each time by 2 CF_2_ groups.^[39]^

Therefore, we elucidated the crystal structures for all 6 target molecules by a combination of single crystal and NMR crystallography.^[40]^ NMR crystallography features the combination of solid‐state NMR spectroscopy together with simulations on the density functional theory (DFT)^[41]^ level to assist Rietveld refinements^[42]^ of powder X‐ray diffractograms. This approach showed to be the method of choice to solve the crystal structure of trisamides,^[43]^ cyclohexane bisamides^[44]^ and many other supramolecular^[45]^ and inorganic^[46]^ systems. All these systems exhibit inherently high defect concentrations and disorder^[47]^ making this combined approach necessary. The crystal structures were correlated to the morphology of the supramolecular nanoobjects and the polarities of the dominantly exposed surfaces were investigated.

## 2 Results and Discussion

Two series of 1,4‐benzene bisamides (Figure 1) were synthesised based on 1,4‐diaminobenzene as core and different perfluorinated carboxylic acids or pivalic acid as side groups. Details on the synthesis and characterization are given in the supporting information (Section 1). The first series (**1** **A**–**1** **C**) comprises a symmetric side group pattern containing linear perfluorinated alkyl chains with increasing length. In contrast, the second series (**2** **A**–**2** **C**) consists of an asymmetric side group pattern with one bulky *t*‐Bu group and one linear perfluorinated alkyl chain with increasing length.

### 2.1 Crystal Structure Solution

By recrystallization from methanol single crystals could be obtained for **1** **A**, **2** **A** and **2** **B** (Figure 1). *Ab initio* structure solution based on direct methods resulted in triclinic (**1** **A**) and monoclinic (**2** **A**, **2** **B**) metrics with R_all_ values of 8.3 % (**1** **A**), 20.1 % (**2** **A**) and 7.6 % (**2** **B**). The centrosymmetric compound **1** **A** crystallises with one half of a molecule in the asymmetric unit in space group *P* $\bar{1}$ . Both **2** **A** and **2** **B** crystallise with one molecule in the asymmetric unit. As the space group of **2** **A** (*P*2_1_/*n*) is centrosymmetric, four molecules are positioned in the unit cell. **2** **B** crystallises in a smaller unit cell with two molecules accounting for the asymmetric space group *P*2_1_. Further crystallographic data of the corresponding structure solutions are listed in Table S2 and in the CIF files deposited in the CSD (compare SI). The phase purity of **1** **A**, **2** **A** and **2** **B** were confirmed by Rietveld refinements of the powder X‐ray diffractograms (PXRD) (Table S2, Figures S2–S4). In particular, for **2** **A**, where the single crystals were extremely anisotropic (thin platelets), the good agreement between the observed and calculated PXRD underlines the structure solution.

As crystallisation experiments for compounds **1** **B**, **1** **C** and **2** **C**, yielded only microcrystalline powders, a combined crystallographic approach with multinuclear (^1^H, ^13^C, ^15^N and ^19^F) solid‐state NMR spectroscopic experiments and computational modelling was applied to compensate for the intrinsic loss of information when solving the crystal structure based on powder samples.

**Figure 2:**
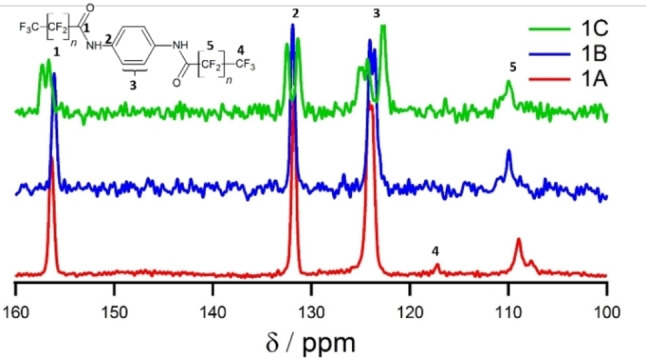
^13^C{^1^H} CP MAS NMR spectra of **1** **A**, **1** **B** and **1** **C**. The carbon atoms of the fluorinated side chains are not well visible as in the CP sequence the polarisation is transferred from the protons to the carbon atoms.

**Figure 3:**
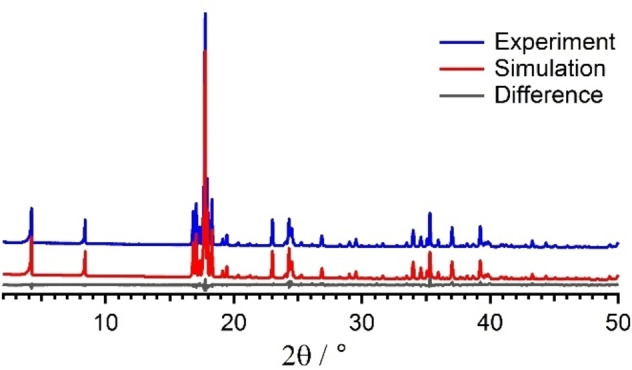
Rietveld profile plot for **1** **B**. The R_wp_ value is 6.0 % and relevant refinement parameters are given in Table S2. The Rietveld plots of the other compounds are displayed in Figures S2–S4.

The ^1^H, ^13^C{^1^H} CP and ^15^N{^1^H} CP magic‐angle‐spinning (MAS) NMR spectra (Figures 2, S5–S8) of compound **1** **B** are very similar in both number and position of the observed resonances compared to the spectra acquired for **1** **A**. This is also true for the powder diffractogram of **1** **B** (Figures 3 and S2). In accordance with the structure solution of **1** **A**, the NMR spectra confirm one half of a molecule in the asymmetric unit for **1** **B** as well. For example, only one ^13^C NMR resonance was observed for the amide unit (Figure 2). Indexing of the PXRD (Figure S4) yields a very similar metric except for a 5 Å longer *c*‐axis, which can be attributed to the larger space requirement due to the increasing length of the perfluorinated side chains. These results suggest an isomorphic structure differing only by the additional two CF_2_‐units in the side chains. Therefore, we extended the structure model of **1** **A** by adding two CF_2_ groups for the structure refinement of **1** **B**. Before the Rietveld refinement, the structure model was geometry optimized with DFT methods. The calculated PXRD of the obtained model is in good agreement with the measured one. (Figure 2 and Table S3). This confirms that **1** **A** and **1** **B** are isomorphic.

The powder pattern of **1** **C** looks very similar to the ones observed for **1** **A** and **1** **B** (Figure S3). The obtained metrics are again very similar and the *c*‐axis is elongated by another 6 Å to fit the space requirements of the additional CF_2_ groups. For the *a‐* and *b*‐axes similar lengths as for **1** **A** and **1** **B** or integer multiples thereof were observed. Although this prevented an unambiguous structure solution for an *ab initio* structure solution, the results imply that **1** **C**, again, is isomorphic to **1** **A** and **1** **B**. Since the ^13^C CP MAS NMR spectrum of **1** **C** exhibits a splitting of all resonances, the space symmetry is reduced to *P*1, probably due to arbitrary torsions of the side chains.

**Figure 4:**
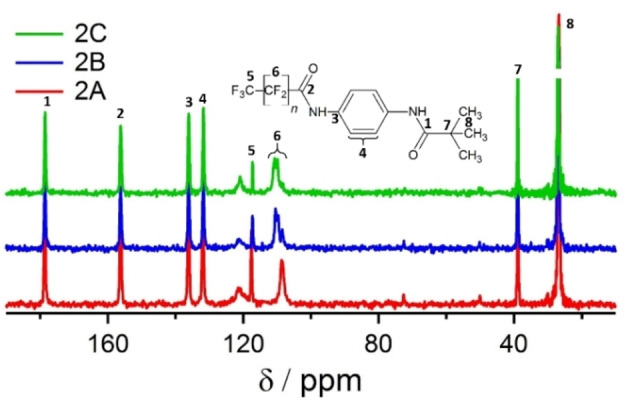
^13^C{^1^H} CP MAS NMR spectra of **2** **A**, **2** **B** and **2** **C** at. The carbon atoms of the fluorinated side chains are not well visible as in the CP sequence the polarisation is transferred from the protons to the carbon atoms.

For series **2**, all NMR spectra (Figures 4 and S5‐S8) have a high level of resemblance, indicating that also **2** **C** crystallises in a unit cell with one molecule in the asymmetric unit. Furthermore, this high degree of consistency between the spectra strongly suggests a very similar local environment. Especially, the identical proton shifts (Figure S5) indicate a uniform hydrogen bond pattern. Indexing the powder diffractogram of **2** **C** leads to a very similar metric compared to **2** **B** with only the *c*‐axis being elongated to account for the increased space requirement of the longer CF_2_‐chain. Therefore, the structure model of **2** **B** was used as a starting point for the structure model of **2** **C**. The lattice parameters were adapted to the ones obtained from the PXRD and two CF_2_‐groups were added to the structure model. The obtained model was geometry optimized with DFT calculations and afterwards refined with Rietveld methods. Again, a very good agreement of the calculated and measured diffractograms was achieved (Figure S4 and Table S2).

To sum up, using single crystal refinements and NMR crystallography in a conjunctive way the crystal structures of **1** **A**, **1** **B** and **2** **A**–**2** **C** were obtained. The crystallographic data of all compounds is deposited in the CSD (compare SI). For **1** **C**, a unit cell is obtained and the NMR spectra together with the unit cell parameters suggest an isomorphic structure, albeit with lower symmetry as for **1** **A** and **1** **B**.

### 2.2 Self‐Assembly and Packing Patterns

**Figure 5:**
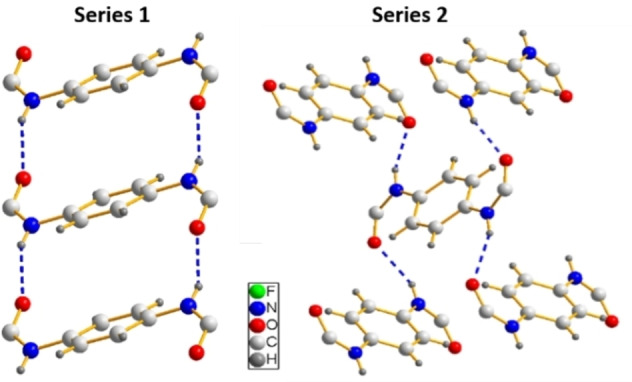
Hydrogen bond arrangement of series **1** (left) and series **2** (right).

Each series of bisamides shows a characteristic hydrogen bond arrangement, which is depicted in Figure 5. For series **1**, one molecule binds to two neighbouring molecules with two hydrogen bonds each, one as a donor and one as an acceptor. This corresponds to the supramolecular synthon observed in references[^30^, ^31^, ^32^, ^33^] Thereby the molecules form a ribbon along the crystallographic *a*‐axis. Along the *b*‐axis, the molecules assemble *via* van der Waals interactions by the interlocking side chains. Along the *c*‐axis only weak van der Waals interactions are detected. Within series **2** one molecule builds four hydrogen bonds to four other molecules as reported within references.[^34^, ^35^, ^36^, ^37^] This leads to a fence‐like crossing pattern with the hydrogen bonds propagating along the *b*‐axis. Along the *a*‐axis the molecules stack in a zig‐zig fashion and along the c‐axis, as for series **1**, only van der Waals interactions are present. A simplified representation of the hydrogen bond patterns is depicted in Figure S9 in the Etter notation.^[25]^

**Figure 6:**
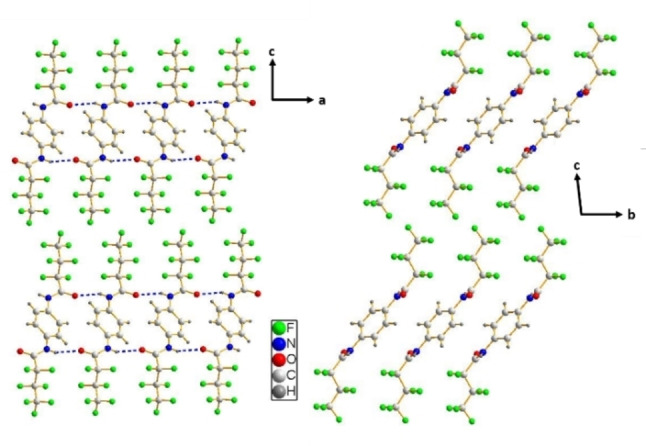
Crystal structure of **1** **A**. On the left the a/c plane is visualised. The hydrogen bond along the a‐axis is visible. On the right the b/c‐plane is depicted. The interlocking CF‐chains along the b‐axis are visible.

**Figure 7:**
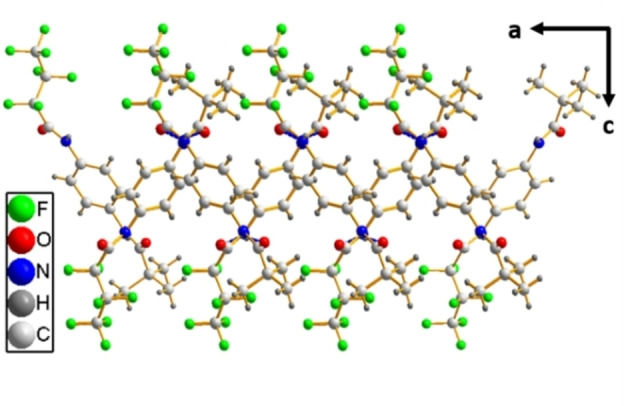
Crystal structure of **2** **A**. The a/c plane is depicted. The propagation of the zig‐zag pattern along the a‐axis is visible.

The local packing of both series lead to layered crystal structures in all cases. The hydrogen bonded ribbons along the a‐axis are the basis of the layers of series **1**. The ribbons stack to each other by van der Waals interactions along the *b*‐axis (Figure 6). For series **2**, the layers consist of molecules in a zig‐zag arrangement along the *a*‐axis and hydrogen bonds along the *b*‐axis (Figure 7). The different orientations of these layers to each other result in different space groups within series **2**. When viewing along the *c*‐axis, all molecules of **2** **B** and **2** **C** are oriented in the same direction, whereas for **2** **A** adjacent molecules in *c*‐direction alter their orientation. This introduces an inversion centre, that leads to the doubling of the *c*‐axis and therefore to 4 molecules in the unit cell. For a graphical explanation, see Figure S10.

These results raise the question why the two series exhibit different packing patterns although their chemical structure is not that different. To investigate this, all crystal structures were geometry optimized on DFT level. For **1** **C**, a model created from **1** **B** was used, as no final crystal structure could be obtained. Furthermore, models were built for each compound in the respective packing pattern of the compound of the other series. For example, a model of **1** **A** was built in the crossed packing pattern of series **2** and so on. For placing the asymmetric molecules of series **2** in the structure type of series **1** several options arise (for more details please refer to SI section 7). For the following discussion, we chose the model with the smallest number of parameters (model 1 in Figure S11) in order to remain comparable to the other set of calculations. These models were geometry optimized on DFT level. The unit cell parameters were allowed to relax, to account for the different space requirements of the molecules.

For all models the dispersion corrected final energy, the lengths of all hydrogen bonds and the densities were extracted (Tables 1 and 2.). The experimentally observed crystal structure is favoured for all compounds by roughly 20 kJ mol^−1^ per molecule.

**Table 1:** Summary of the hydrogen bond lengths and densities obtained from the geometry optimisation on DFT level for all compounds in the packing type of series **1**.

	Packing 1		
	CF−**H**−**O** [Å]	tBu−**H**−**O** [Å]	Density [g cm^−3^]
**1** **A**	1.96	n.a.	2.002
**1** **B**	2.01	n.a.	2.040
**1** **C**	2.03	n.a.	2.131
**2** **A**	2.39	2.41	1.587
**2** **B**	2.47	2.49	1.694
**2** **C**	2.31	2.31	1.755

**Table 2:** Summary of the hydrogen bond lengths and densities obtained from the geometry optimisation on DFT level for all compounds in the packing type of series **2**.

	Packing 2		
	CF−**H**−**O** [Å]	tBu−**H**−**O** [Å]	Density [g cm^−3^]
**1** **A**	1.91^[a]^	n.a.	1.916
**1** **B**	1.91	n.a.	2.023
**1** **C**	2.05^[a]^	n.a.	2.061
**2** **A**	1.82	2.06	1.577
**2** **B**	1.80	2.03	1.648
**2** **C**	1.80	2.02	1.752

[a] averaged.

For the packing type of series **1**, the hydrogen bond lengths for the three molecules crystallising in this packing type (**1** **A**–**1** **C**) increases moderately form 1.96 Å (**1** **A)** to 2.03 Å (**1** **C**). In the same packing type, the hydrogen bond lengths of **2** **A**–**2** **C** vary between 2.31 and 2.48 Å (Table 1). This demonstrates the sensitivity of this packing pattern towards the steric demand of the side groups.

In contrast, the hydrogen bond lengths for the packing type of series **2** shows significantly less deviation, regardless if it's the native packing pattern of this compound or not. For **1** **A** and **1** **B** the average hydrogen bond lengths vary only between 1.91–1.94 Å; **1** **C** exhibits a slightly longer hydrogen bond of 2.05 Å. For **2** **A**–**2** **C** the hydrogen bond next to the CF‐chains range between 1.80 and 1.82 Å, whereas the hydrogen bonds on the other side of the molecule vary between 2.02 and 2.06 Å (Table 2). The hydrogen bond lengths of the symmetric molecules **1** **A**–**1** **C** is the average value of the hydrogen bond lengths of the asymmetric molecules **2** **A**–**2** **C** in the packing type of series **2**. This demonstrates the robustness of this packing type towards the steric demand of the side group.

These observations are in line with the trend of the ^1^H chemical shifts (Figure S5). For series **1** the increase of the hydrogen bond lengths from **1** **A** to **1** **C** translates into a decreasing chemical shift of the N−H protons from 9.5 to 9.1 ppm. The equivalent N−H protons of series **2** (next to the CF‐chain) resonate at 10.7 ppm, independent of the chemical composition of the individual compound. The higher shift for series **2** indicates a shorter and thus stronger hydrogen bond for this series.

These findings demonstrate that **1** **A**–**1** **C** crystallise in their native packing pattern, although slightly shorter hydrogen bonds could be achieved when crystallizing in packing type of series **2**. Apparently, within this series, the loss in hydrogen bond strengths is over compensated by a gain in van der Waals interactions due to a denser packing of the molecules in packing type of series **1** (Tables 1 and 2). Also, for **2** **A**–**2** **C** the calculated densities (Tables 1 and 2) is higher when crystallising in packing type of series **1**, promising a gain in van der Waals interactions. However, due to the steric demand of the side groups, only weak hydrogen bonds, that are 0.6 Å longer compared to the native structure, could be realised. This loss in hydrogen bond strength cannot be compensated by the gain in van der Waals interactions when crystallising in the packing type of series **1**.

From this, we identified two coupled driving forces (density, hydrogen bond strength) that determine the crystal structure of benzene bisamides. On the one hand, a dense packing is favoured on the other hand, this should not increase the hydrogen bond length markedly. For **1** **A**–**1** **C**, the slim CF chains allow for a dense packing with one molecule forming four hydrogen bonds to two neighbouring molecules with short hydrogen bonds. For **2** **A**–**2** **C**, the bulky *t*‐Bu groups prevent short hydrogen bonds in the same arrangement. Therefore, the molecules rotate and the central molecule forms four hydrogen bonds to four other molecules.

A comparison with published structures from the literature are in agreement with these findings. For example, Dhamodrahn et al. published a structure with a bulky 1‐brom‐1‐methyl‐ethyl side group, which crystallises in the crossed packing of series **2**.^[35]^ The same is true for the structure where both side groups contain *t*‐butyl.^[36]^ Whereas, Mangalugui et al. published the crystal structure of a bisamide with a slim 3‐chloro‐propyl side chain, which assembles in the parallel packing of series **1**.^[32]^ A bisamide with cyclohexane side groups is also slim enough to crystallise in the packing type of series **1**.^[17]^ For very bulky side groups, like leucine derivatives, the structure opens up further and builds a so called nano‐staircase.^[34]^ These results show how the crystal structure of a 1,4‐benzene bisamide derivatives can be tailored by a thoughtful choice of the peripheral substitution pattern. We thus identified the bulkiness of the side group as the molecular motif that directs the crystal structure.

### 2.3 Morphology of the Self‐Assembled Nano‐objects

To probe the influence of the anisotropic interactions between the molecules within the crystal structure, we investigated the self‐assembly behaviour to nanoobjects of compounds **1** **A**–**1** **C** and **2** **A**–**2** **C** in solution. Since self‐assembly to a well‐defined morphology typically depends not only on the molecular structure but also on the applied temperature protocol, solvent, and concentration of the compounds, we have evaluated these process parameters. We found that by selecting o‐dichlorobenzene as solvent, all 1,4‐benzene bisamides can be molecularly dissolved at a temperature of 120 °C at a concentration of 500 ppm. Upon rapid cooling, the initially clear solution turned turbid indicating the formation of solid objects. These dispersions were drop casted onto silicon wafers and investigated by means of scanning electron microscopy (SEM).

**Figure 8:**
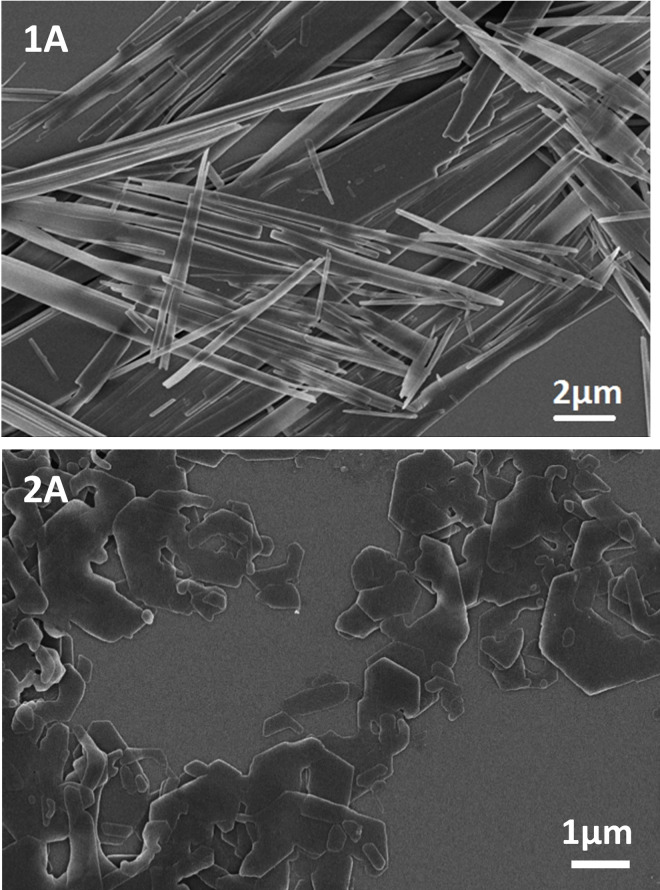
SEM images of platelets of **1** **A** (top) and of **2** **A** (bottom).

Figure 8 shows exemplarily SEM micrographs of the anisotropic morphology obtained from **1** **A** and **2** **A**. The SEM micrographs of **1** **B**–**1** **C** and **2** **B**–**2** **C** are given in Figures S11 and S12. All molecules form apparently thin 2D structures, however, with distinct differences in their morphology. For instance, all structures formed by the symmetrically substituted 1,4‐benzene bisamides (**1** **A**–**1** **C**) feature a defined ribbon‐like shape. In contrast, all structures formed by the asymmetrically substituted 1,4‐benzene bisamides (**2** **A**–**2** **C**) feature platelet‐like shapes with an irregular body. Moreover, in series **1** the width of the ribbons increases with increasing size of the perfluorinated side groups. In contrast, the diameter of the platelets of series **2** decreases with increasing size of the perfluorinated side groups.

These findings fit very well to the crystallographic data, where each series self‐assembles into one distinct packing pattern. For instance, in series **1** the hydrogen bond pattern propagates along the crystallographic *a*‐axis, whereas along the other two axes only weak van der Waals interactions are effective. During the self‐assembly, this results into the anisotropic nanostructrures featuring a preferred growth rate in one direction. Along the crystallographic *b*‐axis, the close packing of the CF‐chains leads to a stronger van der Waals interaction compared to the one present along the *c‐*axis. With increasing number of CF_2_ groups, the former becomes stronger while the latter remains unchanged. This explains the ribbon‐like structure with increasing widths of the ribbons when the length of the perfluorinated side chain increases.

In contrast, for series **2** we derived two directions of favoured growth from the crystal structure. Because of the zig‐zag‐pattern of the hydrogen bond motif along the *a‐* and *b‐*axes, the platelet like morphology observed for all self‐assembled nanoobjects of series **2** is explained. The platelets, prepared under identical conditions, become smaller with increasing lengths of the perfluorinated chains. We attribute this to the geometrical mismatch of the perfluorinated and the alkyl side chains that alternate along the *a*‐axis. This results in the formation of smaller platelets when the perfluorinated side chain increases.

**Figure 9:**
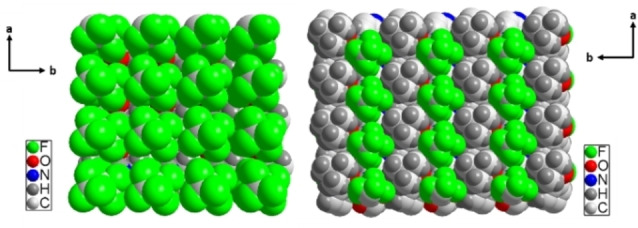
Surfaces of series **1** (left) and series **2** (right).

Atomic force microscopic (AFM) images (Figure S13) of the self‐assembled nanoobjects show distinct terraces for all compounds. The height of these terraces is in all cases in the order of the lengths of the corresponding crystallographic *c*‐axis of the respective bisamides (1/2 *c* for **2** **A**) implying that the terminating face of the macroscopic structures is an all cases the (001) face. This interpretation is in line with the discussion of the growth rates. For series **1** the crystal growth mainly propagates along the *a*‐axis and secondary along the *b*‐axis. This leaves the perfluorinated (001) face (Figure 9 left) as the terminating surface of the ribbons, since this face has the lowest tendency to grow further. For series **2** crystal growth propagates along the *a*‐ and *b‐*axes leading to a surface that has alternating rows of CF_3_ and *t‐*Bu end groups (Figure 9 right).

**Figure 10:**
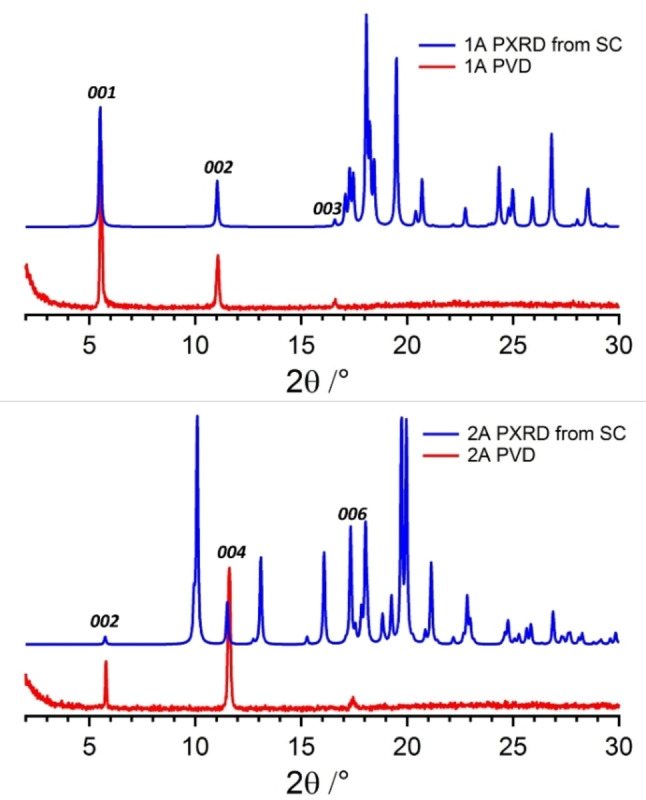
Simulated X‐ray diffractograms from the single crystal structure (blue) and measured X‐ray diffractograms of a the deposited samples (red) of **1** **A** (top) and **2** **A** (bottom).

Due to the different composition of the surfaces, different polarities are expected. Since the nanoobjects are too small to be directly measured by contact angle measurements, we deposited **1** **A** and **2** **A** on silanised glass wafers by physical vapour deposition. X‐ray diffraction measurements on the resulting thin films show that in both cases only reflexes of the *(00l)* series (Figure 10) remain proving the successful deposition of an oriented thin film with the crystal structure of the corresponding bulk phases and a terminating (001) face. For **2** **A**, where two layers of molecules are present in the *c‐*direction of the crystal structure the symmetry is broken when depositing the material on the glass wafer. Therefore, only even numbers of *l* are present in the diffractogram since these reflexions correspond to the layer spacing.

On these glass wafers, water/air contact angle measurements were performed (Figure S14). The measurements on the film of **1** **A** yielded an average contact angle of 115°, that is in the range of neat Teflon films,^[48]^ showing that this film is highly hydrophobic. For the film of **2** **A**, a less hydrophobic average contact angle of 105° was obtained, which is in line with the lower amount of fluorine on the surface due to alternating arrangement of *t‐*Bu and CF_3_ groups. Still this contact angle is higher compared to purely aliphatic surfaces.^[49]^

We are thus able to predict and tune the resulting morphology and surface polarities of the self‐assembled nanoobjects based on the crystal structure, which itself can be predicted by the molecular structure.

## 3 Conclusion

The crystal structures of two series of 1,4‐benzene bisamides with symmetric and asymmetric peripheral substitution were solved using a combination of single crystal diffraction and NMR crystallography. The symmetric series **1** consists of two linear, perfluorinated alkyl chains of increasing length (2/4/6 CF_2_ units). The three compounds of series **1** crystallise in the same packing type, where one bisamide molecule forms four hydrogen bonds to two neighbouring molecules (Figure 5). These molecules are arranged parallel and thus create ribbons. These ribbons are densely packed along the other two crystallographic directions (Figure 6). For the asymmetric series **2** one side chain is substituted by a *t*‐butyl group. The compounds of this series prefer a second packing type, where one bisamide molecule expresses four hydrogen bonds to four other molecules (Figure 5). These molecules are arranged in a zig‐zag fashion creating a 2D layer (Figure 7). These layers are stacked in the third crystallographic direction.

SEM micrographs of the self‐assembled nanoobjects showed that series **1** crystalises in thin ribbons, whereas series **2** self‐assembles into thin platelets. Thus, for both series the molecular self‐assembly translates into a distinct anisotropic morphology of the resulting objects. Contact angle measurements on thin films, vapour deposited on silanised glass wafers, demonstrate that the exposed surfaces are markedly hydrophobic, with series **1** exceeding the hydrophobicity of series **2**. The X‐ray diffraction measurements of these films (Figure 10) showed that in both cases the (001) face is exposed, which coincides with the dominantly exposed surface of the self‐assembled nanoobjects. The (001) surface consists of CF_3_ groups for series **1** and of alternating rows of *t*‐butyl and CF_3_ groups for series **2**, explaining the different wetting behaviour for both series.

Based on DFT calculations, we identified two interacting driving forces – the hydrogen bond strength within the amide core and the crystal density – that, when tailored, allow to switch between the two packing patterns. Slim side chains like perfluorinated alkyl groups, favour dense packings without reducing the hydrogen bond strength in either of the packing types and packing pattern of series **1** with its 1D preferentially crystal growth is energetically favoured. Bulky side groups like *t*‐butyl groups, weaken the hydrogen bonds at the bisamide core if ribbons would form. Therefore, they prefer packing type of series **2**, in spite of its lower density.

Essentially, we show that the steric demand of the peripheral substitution of 1,4‐subsititued benzene bisamides can be used as a leitmotif to programme the crystal structure which in turn guides the crystal growth due to the spatial anisotropy within the interaction strength. This allows for tailoring the crystal morphology and the surface properties like polarity of bisamide nanoobjects and will be beneficial for identifying promising candidates for applications such as polymer additives in the future.

## Experimental Section

Details on the synthesis, characterisation and temperature of 5 % weight loss (Table S1) of the symmetric and asymmetric 1,4‐bisamides are given in the supporting information (Section 1).

Self‐assembly experiments were performed by dissolving 500 ppm of the respective 1,4‐benzene bisamide in 2.5 mL of o‐dichlorobenzene in a 4 mL glass vial with screw cap and subsequent rapid cooling of the solution in an ice bath for 15 min.

Single crystal X‐ray diffraction experiments were carried out on a STOE IPDS II instrument (Mo‐K_α_ radiation) equipped with a Ge(111) monochromator at 173 K. The crystals were mounted on a glass tip with glue. Data collection, indexing, space group determination, data reduction and reconstruction of reciprocal space layers were performed with the software package X‐Area (Stoe). Structure solution was carried out with the software package Shelx.^[50]^

Powder X‐ray diffraction measurements were carried out on a STOE STADI P diffractometer equipped with a Ge(111) monochromator using Cu‐K_α1_ radiation. The powders were filled in 0.5 mm or 0.3 mm capillary tubes and were measured in Debye‐Scherrer geometry under ambient conditions or at 173 K. Indexing, simulated annealing and Rietveld refinement were done with the software package TOPAS.^[51]^ Models were built with Materials Studio.

X‐ray diffraction patterns of thin films were obtained using nickel filtered Cu‐K_α_ radiation on a Bragg‐Brentano‐type diffractometer (XPERT‐PRO, PANalytical B.V) equipped with an X'Celerator Scientific RTMS detector.

NMR spectroscopic experiments were performed on a Bruker Avance III 600 NMR Spectrometer (600.15 MHz). ^1^H and ^19^F experiments were performed in a 1.3 mm ZrO_2_ rotor at 62.5 kHz MAS frequency. ^13^C {^1^H} CP and ^13^C {^19^F} CP experiments were carried out with a 3.2 mm rotor at 16 kHz. The CP experiments were done with a ramped CP sequence^[52]^ where the nutation frequency on the ^1^H or ^19^F channel was varied linearly from 50–100 %. The maximum nutation frequencies during the contact time were set to 87 kHz (^1^H) and 95 kHz (^19^F), respectively. During acquisition on the ^13^C channel, ^1^H and ^19^F were decoupled using the Spinal64^[53]^ decoupling sequence with nutation frequencies of 92 and 89 kHz, respectively. ^15^N {^1^H} CP NMR spectroscopic experiments were performed on a Bruker Avance III 400 NMR Spectrometer (400.13 MHz). Samples were packed in 3.2 mm Zirconia rotors and measured at 10 kHz MAS frequency. The same ramped CP sequence as above was applied with a maximum proton nutation of 70 kHz but only protons were decoupled with Spinal64 at 75 kHz.

Scanning electron microscopy: Prior to the investigation, the dispersion of the samples were drop casted on a silicon wafer and dried. Subsequently, the samples were sputtered with a platinum layer with a thickness of 1.3 nm using a Cressington 208HR sputter coater. SEM was performed with a field emission scanning electron microscope (Zeiss LEO 1530) using an acceleration voltage of 3 kV.

Physical vapor deposition (PVD) was performed using a custom‐made vacuum chamber (Balzers PLS 500) equipped with three effusion cells. The evaporation setup is described in detail in the literature.^[54]^ Prior to the evaporation, glass substrates were silanized with HDMS applying a standard procedure. The vacuum chamber was evacuated to 4×10^−6^ mbar and the effusion cells were heated to 130 °C for **1** **A** and 105 °C for **2** **A** during the evaporation. A constant evaporation rate of about 0.3 Ås^−1^ was used. The evaporation time was typically 60 min resulting in film thickness of about 100 nm.

Atomic force microscopy (AFM) measurements were performed using a Veeco dimension 3100 atomic force microscope equipped with a NanoScope IV controller. Bruker OTESPA‐R3 silicon cantilevers were used in tapping mode. Images were evaluated using Bruker NanoScope Analysis software (version 1.40).

Contact angle measurements were performed on vapour deposited thin films with the sessile drop method using a Krüss DSA25S drop shape analyser. For each thin film, the average contact angle of at least five measurements was determined.

For the quantum mechanical calculations the structures obtained from single crystal and powder X‐ray refinement were geometry optimised^[55]^ on DFT level with the software package CASTEP^[56]^ using the PBE functional and the Tkatchenko‐Scheffler^[57]^ dispersion correction scheme. An electronic cut off energy of 900 eV and a Monkhorst k point grid spacing of 0.07 Å^−1^ was used.^[58]^ Force field calculations were done with the DREIDING^[59]^ force field and charges were applied using Gasteiger.^[60]^

This work was financially supported by the German Research Foundation (DFG), project number 79971943 (Collaborative Research Center SFB 840, project B4). KvdZ thanks the Elite Study Program Macromolecular Science within the Elite Network of Bavaria (ENB) for support. The authors would like to thank Dr. Tiziana Boffa‐Ballaran (Bavarian Geoinstitute), Dr. Wolfgang Milius and Dr. Thomas Martin (Inorganic Chemistry I, UBT) for fruitful discussions on crystallography. Markus Stihl (Macromolecular Chemistry I, UBT) is gratefully acknowledged for preparing thin films by physical vapor deposition. Sandra Ganzleben (Macromolecular Chemistry I, UBT) is thanked for the synthesis of the molecules.

## Conflict of interest

The authors declare no conflict of interest.

## Supporting information

As a service to our authors and readers, this journal provides supporting information supplied by the authors. Such materials are peer reviewed and may be re‐organized for online delivery, but are not copy‐edited or typeset. Technical support issues arising from supporting information (other than missing files) should be addressed to the authors.

Supporting Information
