# Supplementary material for: Crystal Engineering of Supramolecular 1,4‐Benzene Bisamides by Side‐Chain Modification – Towards Tuneable Anisotropic Morphologies and Surfaces
Source: Chemphyschem. 2021 Nov 8;22(24):2585–93. doi: 10.1002/cphc.202100597 (PMC9299472; doi:10.1002/cphc.202100597)
Supplement: Supplementary file 1 — Supporting Information [file CPHC-22-2585-s001.pdf]

ChemPhysChem

Supporting Information

Crystal Engineering of Supramolecular 1,4-Benzene Bisamides by Side-Chain Modification – Towards Tuneable Anisotropic Morphologies and Surfaces

Kasper P. van der Zwan, Christoph Steinlein, Klaus Kreger, Hans-Werner Schmidt,* and Jürgen Senker*

Table of content

1: Synthesis and characterization of symmetric 1,4-benzene bisamides **1A**-**C** and asymmetric 1,4-benzene bisamides **2A**-**C** 2

2: Crystallographic information of the single crystal structure solutions 6

3: Powder X-ray diffractograms with Rietveld refinements 8

4: Additional MAS NMR spectra of symmetric 1,4-benzene bisamides **1A**-**C** and asymmetric 1,4-benzene bisamides **2A**-**C** 9

5: Different coordination of the two series 10

6: Different global packing of **2A** compared to **2B** and **2C.** 10

7: Structure models of **2A** in the structure type of series **1** 11

8: Scanning electron microscopy of self-assembled platelets of symmetric 1,4-benzene bisamides **1B**-**C** and asymmetric 1,4-benzene bisamides **2B**-**C** 13

9: AFM of self-assembled platelets of both series 14

10: Thin film preparation and wetting behavior with water on vapor deposited thin films of symmetric 1,4-benzene bisamides **1A** and asymmetric 1,4-benzene bisamides **2A** 16

1: Synthesis and characterization of symmetric 1,4-benzene bisamides **1A**-**C** and asymmetric 1,4-benzene bisamides **2A**-**C**

Synthetic route to symmetrically subsituted 1,4-bisamides

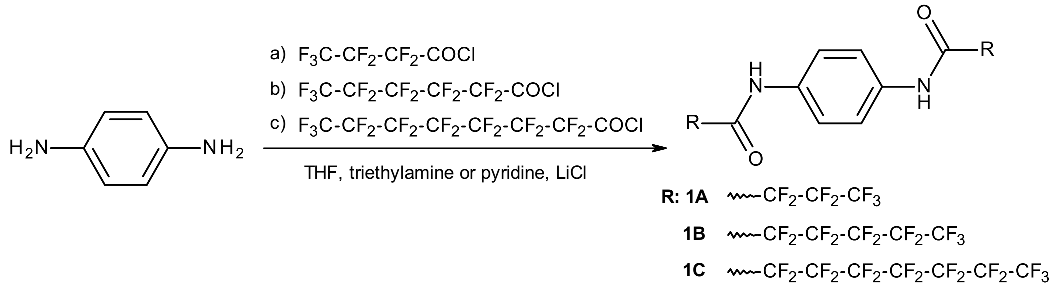

Synthetic route to asymmetrically subsituted 1,4-bisamides

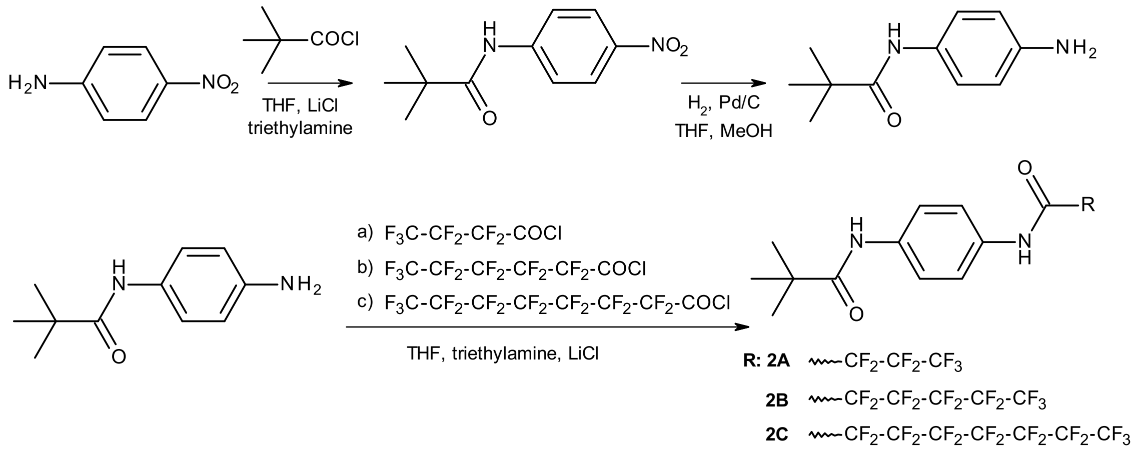

Figure S1. Top: One step synthesis to symmetric 1,4-bisamides **1A**-**C** by conversion of 1,4-diaminobenzene with the respective perfluorinated acid chlorides. Bottom: Three step synthesis to asymmetric 1,4-bisamides **2A**-**C**: Conversion of 4-nitroaniline with pivaloyl chloride, hydrogenation of 2,2-dimethyl-N-(4-nitrophenyl)propanamide and subsequent conversion of 2,2-dimethyl-N-(4-aminophenyl)propanamide with the respective perfluorinated acid chlorides.

Synthesis of N,N'-1,4-phenylenebis(2,2,3,3,4,4,4-heptafluorobutanamide) ***1A***

4.7 g of perfluorobutanoyl chloride was added to a mixture of 1.0 g of 1,4-phenylenediamine, 3 mL of pyridine, a small amount of LiCl and 100 mL of anhydrous THF at 0 °C under argon atmosphere. The reaction mixture was heated to 40 °C for 12 h. After evaporation of the solvent, the solid residue was dispersed in water and filtered off. Crystallization from methanol yielded 4.4 g (94%) of the product as white crystals.

^{1}H NMR (300 MHz, DMSO-d_6) δ (ppm): 11.40 (2 H, s), 7.71 (4 H, s).

MS-EI (m/z, %): 500 ([M]+, 100), 481 (31), 331 (15), 303 (98), 108 (65), 81 (15), 69 (23).

Synthesis of N,N'-1,4-phenylenebis(2,2,3,3,4,4,5,5,6,6,6-undecafluorohexanamide) ***1B***

5.3 g of perfluorohexanoyl chloride were slowly added to a mixture of 0.66 g of 1,4-phenylenediamine, 3 mL of pyridine, a small amount of LiCl and 100 mL of anhydrous THF at 0 °C under argon atmosphere. The reaction mixture was refluxed for 12 h. After solvent removal, the solid residue was dispersed in water and filtered off. Crystallization from methanol yielded 1.8 g (43%) of the product as white solid.

^{1}H NMR (300 MHz, DMSO-d_6) δ (ppm): 11.39 (2 H, s), 7.69 (4 H, s).

MS-EI (m/z): 700 ([M]+, 100), 681 (10), 431 (13), 303 (95), 133 (12), 108 (38), 69 (11).

Synthesis of N,N'-1,4-phenylenebis(2,2,3,3,4,4,5,5,6,6,7,7,8,8,8-pentadecafluorooctanamide) ***1C***

6.6 g of perfluorooctanoyl chloride were slowly added to a mixture of 0.75 g of 1,4-phenylenediamine, 5 mL of triethylamine, a small amount of LiCl and 200 mL of anhydrous THF at 0 °C under argon atmosphere. Subsequently, the reaction mixture was refluxed for 12 h. After solvent evaporation, the solid residue was dispersed in water and filtered off. Crystallization from N,N-dimethylformamide yielded 5.1 g (81%) of the product as white solid.

^{1}H NMR (300 MHz, DMF-d_6) δ (ppm): 11.68 (2 H, s), 8.20 (4 H, s).

MS-EI (m/z): 900 ([M]+, 100), 881 (47), 531 (6), 503 (55), 133 (6), 108 (28), 69 (11).

Synthesis of 2,2-dimethyl-N-(4-nitrophenyl)propanamide

2.9 g of pivaloyl chloride was added to a mixture of 3.0 g of 4-nitroaniline, 20 mL of trimethylamine and 150 mL of anhydrous THF at 0 °C under argon atmosphere. The reaction mixture was refluxed for 12 h. After removal of the solvents, the solid residue was washed with water and recrystallized from hexane, yielding 4.3 g (89%) of the product.

^{1}H NMR (300 MHz, DMSO-d_6) δ (ppm): 9.79 (1 H, s), 8.21 (2 H, m), 7.95 (2 H, m), 1.25 (9 H, s).

Synthesis of 2,2-dimethyl-N-(4-aminophenyl)propanamide

4.3 g of 2,2-dimethyl-N-(4-nitrophenyl)propanamide were added to 250 mL of THF and 50 mL of MeOH together with 0.4 g palladium on activated charcoal (10% Pd). Hydrogenation was performed with H_2 at a pressure of 3 bar and 35 °C for 12 h. The catalyst was removed by filtration of the reaction mixture over Alox N. After evaporation of the solvent, the product was obtained as white powder in quantitative yield (3.7 g).

^{1}H NMR (300 MHz, DMSO-d_6) δ (ppm): 8.80 (1 H, s), 7.18 (2 H, m), 6.48 (2 H, m) , 4.84 (2 H, s), 1.18 (9 H, s).

Synthesis of N-[4-[(2,2-dimethyl-1-oxopropyl)amino]phenyl]-2,2,3,3,4,4,4-heptafluorobutanamide ***2A***

3.1 g of perfluorobutanoyl chloride were slowly added to a mixture of 2.0 g of 2,2-dimethyl-N-(4-aminophenyl)propanamide, 10 mL of triethylamine, a small amount of LiCl and 100 mL of anhydrous THF at 0 °C under argon atmosphere. Subsequently, the reaction mixture was refluxed for 12 h. After evaporation of solvents, the solid residue was dispersed in water and filtered off. Crystallization from methanol yielded 2.6 g (64%) of the product as white crystals.
^{1}H NMR (300 MHz, DMSO-d_6) δ (ppm): 11.23 (1 H, s), 9.30 (1 H, s), 7.62 (4 H, m), 1.22 (9 H, s)
MS-EI (m/z, %): 388 ([M]+, 72), 304 (29), 107 (17), 85 (15), 57 (100), 41 (12).

*Synthesis of N-[4-[(2,2-dimethyl-1-oxopropyl)amino]phenyl]-2,2,3,3,4,4,5,5,6,6,6-undecafluorohexanamide **2B***
4.5 g of perfluorohexanoyl chloride were slowly added to a mixture of 2.0 g of 2,2-dimethyl-N-(4-aminophenyl)propanamide, 10 mL of triethylamine, a small amount of LiCl and 100 mL of anhydrous THF at 0 °C under argon atmosphere. Subsequently, the reaction mixture was refluxed for 12 h. After evaporation of solvents, the solid residue was dispersed in water and filtered off. Crystallization from methanol yielded 1.0 g (20%) of the product as white crystals.
^{1}H NMR (300 MHz, DMSO-d_6) δ (ppm): 11.23 (1 H, s), 9.30 (1 H, s), 7.62 (4 H, m), 1.22 (9 H, s).
MS-EI (m/z, %): 488 ([M]+, 77.0), 469 (14), 404 (31), 107 (14), 85 (15), 57 (100), 41 (11).

*Synthesis of N-[4-[(2,2-dimethyl-1-oxopropyl)amino]phenyl]-2,2,3,3,4,4,5,5,6,6,7,7,8,8,8-pentadecafluorooctanamide **2C***
4.4 g of perfluorooctanoyl chloride were slowly added to a mixture of 1.5 g of 2,2-dimethyl-N-(4-aminophenyl)propanamide, 10 mL of triethylamine, a small amount of LiCl and 100 mL of dry THF at 0 °C in inert atmosphere. The mixture was refluxed for 12 h. After solvent removal, the solid was dispersed in water and filtered off. Crystallization from ethyl acetate yielded 3.85 g (84%) as white crystals.
^{1}H NMR (300 MHz, DMSO-d_6) δ (ppm): 11.21 (1 H, s), 9.30 (1 H, s), 7.61 (4 H, m), 1.22 (9 H, s).
MS-EI (m/z, %): 588 ([M]+, 27), 569 (8), 504 (14), 107 (11), 85 (15), 57 (100).

For all substances, melting and sublimation points, as determined by DSC and TGA measurements, are too close to each other to be distinguished properly. Therefore, we provide the temperature of 5 % mass loss in Table S1.

Table S1. Temperature of 5 % weight loss.

Compound	Temperature /°C
1A	221
1B	221
1C	221
2A	212
2B	216
2C	235

Analytical characterization methods

Nuclear magnetic resonance spectroscopy: ^{1}H NMR (300 MHz) experiments were carried out on a Bruker Avance AC 300 spectrometer at room temperature. For all compounds solid state ^{13}C NMR spectra are given in Figures 2,4 and S8.

Mass spectroscopy: Mass spectra were recorded on a Finnigan MAT 8500 spectrometer (Thermo Fisher Scientific) (EI, 70 eV) using direct injection mode.

2: Crystallographic information of the single crystal structure solutions

Table S2. Crystallographic information for single crystal structure solutions. The CIF files are deposited in the Cambridge Structural Database with the deposition numbers 2101449, 2101447 and 210144 for **1A**, **2A** and **2B**, respectively.

Compound	1A	2A	2B
Molecular formula	$C_{14}H_6F_{14}N_2O_2$	$C_{15}H_{15}F_7N_2O_2$	$C_{17}H_{15}F_{11}N_2O_2$
M/g mol^{-1}	500.19	388.29	488.3
Radiation	Mo-Kα	Mo-Kα	Mo-Kα
No. of reflections	2269	4120	5351
No. of reflections(I/σ>2)	1557	2192	4938
Theta range	2.54-29.24	2.29-28.28	2.19-29.15
Completeness	99.7	99.5	99.5
h,k,l range	±6, ±7, ±21	±12, ±7, ±40	±12, ±7, ±25
Crystal system	triclinic	monoclinic	monoclinic
Space group	$P\bar{1}$	$P2_1/n$	$P2_1$
a/Å	5.1278(10)	9.2124(18)	9.2340(18)
b/Å	5.2125(10)	5.8994(12)	5.8718(12)
c/Å	16.090(3)	30.732(6)	18.787(4)
α/°	95.27(3)	90	90
β/°	90.74(3)	91.57(3)	98.87(3)
γ/°	91.74(3)	90	90
V/Å^3	428.00(15)	1669.6(6)	1006.5(3)
Z'/Z	0.5/1	1/4	1/2
r/g cm^{-3}	1.940	1.547	1.61
T/ K	173	173	173
Goodness of fit	1.069	1.485	1.124
R_{all}	0.0828	0.2008	0.0764
R (I/σ>2.0)	0.0533	0.1404	0.067
wR_{all}	0.1469	0.35	0.1804
wR (I/σ>2.0)	0.1241	0.3098	0.1708
Residual electron density	0.374 e/Å^3	0.621 e/Å^3	1.04 e/Å^3

Table S3. Crystallographic information for powder X-ray crystal structure solutions. The CIF files are deposited in the Cambridge Structural Database with the deposition numbers 2101446, 2101445, 2101450, 2101448 and 210143 for **1A**, **1B**, **2A**, **2B** and **2C**, respectively.

Compound	1A	1B	1C	2A	2B	2C
Refinement	Rietveld	Rietveld	Pawley	Rietveld	Rietveld	Rietveld
Molecular formula	$C_{14}H_6F_{14}N_2O_2$	$C_{18}H_6F_{22}N_2O_2$	$C_{22}H_6F_{30}N_2O_2$	$C_{15}H_{15}F_7N_2O_3$	$C_{17}H_{15}F_{11}N_2O_2$	$C_{19}H_{15}F_{15}N_2O_2$
M/g mol^{-1}	500.19	700.22	900.25	389.29	488.3	588.31
Crystal system	triclinic	triclinic	triclinic	monoclinic	monoclinic	monoclinic
Space group	$P\bar{1}$	$P\bar{1}$	n.A.	$P2_{1/n}$	$P2_1$	$P2_1$
a/Å	5.11628(6)	5.11602(4)	5.5187(5)	9.2727(5)	9.3315(7)	9.3809(2)
b/Å	5.19633(10)	5.28202(5)	5.7129(5)	6.1044(4)	5.9830(3)	5.9346(1)
c/Å	16.1019(4)	21.1591(3)	27.0745(43)	30.9098(27)	18.7312(18)	22.0028(11)
α/°	95.382(2)	93.976(1)	90.662(9)	90	90	90
β/°	90.723(2)	88.824(2)	92.243(10)	96.440(6)	98.496(4)	106.682(4)
γ/°	91.641(2)	89.255(2)	114.576(5)	90	90	90
V/Å^3	425.97(1)	570.22(1)	775.31(17)	1738.61(1)	1034.31(1)	1173.40(8)
Z'/Z	0.5/1	0.5/1	n.A.	1/4	1/2	1/2
ρ/g cm^{-3}	1.950	2.039	1.928	1.292	1.568	1.66
T/ K	173	293	293	293	293	293
R_p	0.034	0.041	0.029	0.044	0.043	0.035
R_{wp}	0.046	0.060	0.046	0.060	0.060	0.056

A preferred orientation of the 4th order using spherical harmonics [1] has been applied to all models except the Pawley fit.

The single crystal solution of **2A** yielded a cell in the space group $P2_1/n$, however, the R-values are relatively poor. This is explained by the habitus of the crystal. The compound crystalizes in very thin platelets leading to a limited number of strong reflexions. Indexing the PXRD led to a similar but slightly different cell in the space group $P2_1$. The absence of the glide plane leads to a doubling of the asymmetric unit. The single crystal data was refined in this space group as well and the obtained model placed in the cell obtained from PXRD. This model was geometry optimised by force field and DFT methods to correct for the now incorrect bond lengths and angles. The geometry optimized model could be refined with a good agreement with Rietveld methods. Then, the glide plane was found again and the Rietveld refinement could be redone with the space group $P2_1/n$ yielding even better R-values (Figure S3). This shows that although the R-values result in an A-Alert in the CheckCIF report, the structure solution arising from the single crystal is correct and the data quality arises from the desired and consequently engineered morphology as a thin platelet.

[1] M. Järvinen, J Appl Crystallogr 1993, 26, 525.

3: Powder X-ray diffractograms with Rietveld refinements

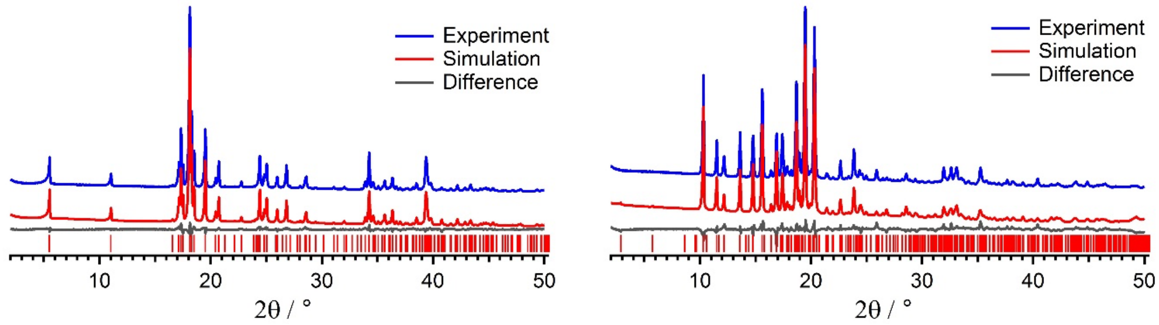

Figure S2. Powder X-ray diffractogram with Rietveld refinement of **1A** (left) and **2A** (right).

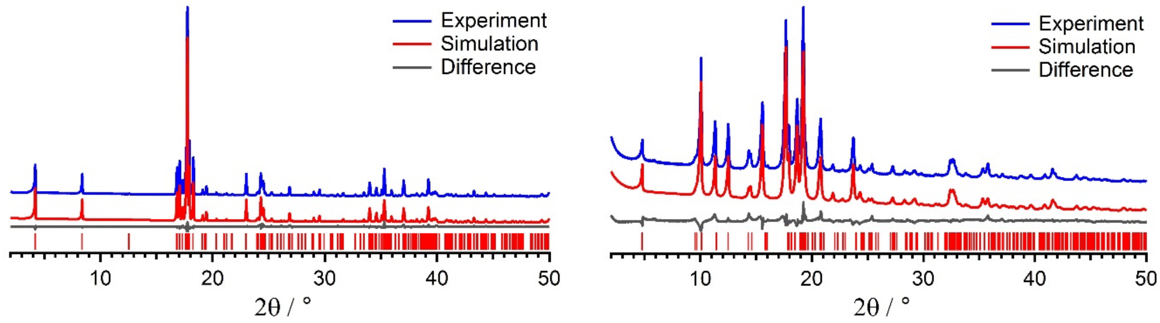

Figure S3. Powder X-ray diffractogram with Rietveld refinement of **1B** (left) and **2B** (right).

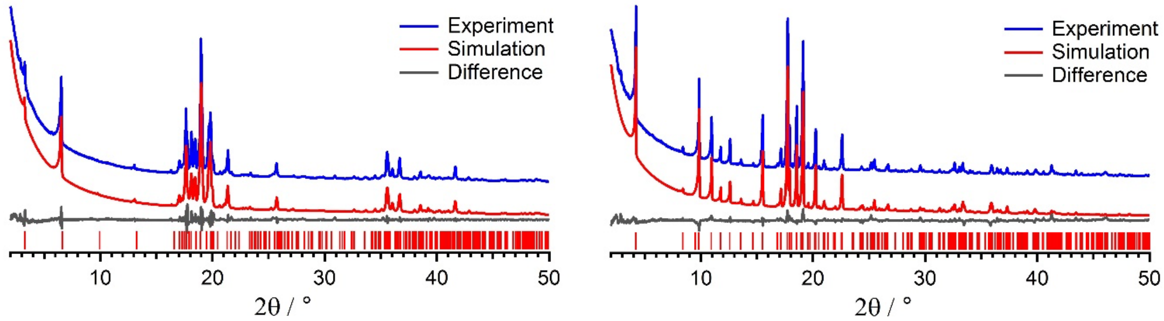

Figure S4. Powder X-ray diffractogram with Pawley refinement of **1C** (left) and with Rietveld refinement of **2C** (right).

4: Additional MAS NMR spectra of symmetric 1,4-benzene bisamides **1A**-**C** and asymmetric 1,4-benzene bisamides **2A**-**C**

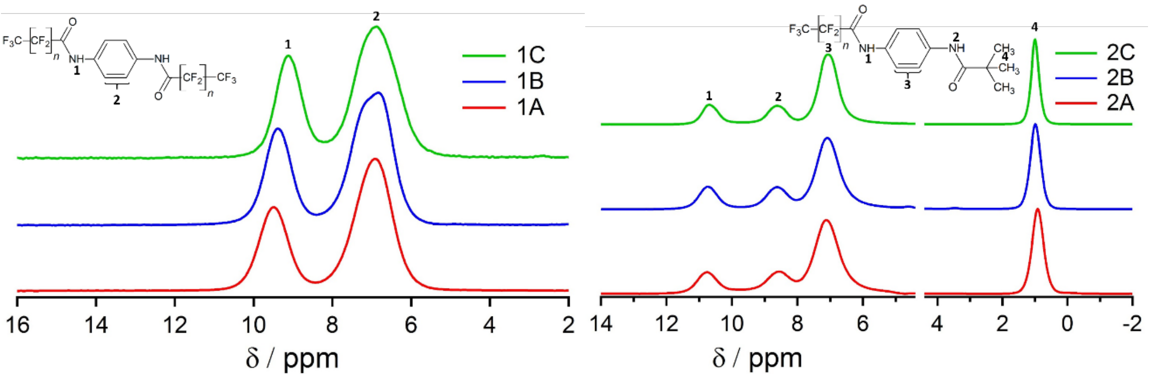

Figure S5. ^{1}H MAS-NMR spectra of series **1** (left) and series **2** (right).

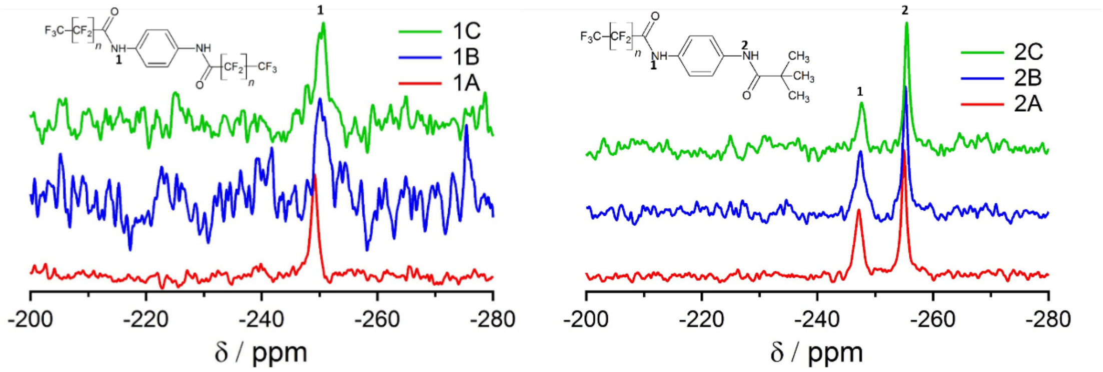

Figure S6. $^{15}N\{^{1}H\}$ CP MAS-NMR spectra of series **1** (left) and series **2** (right).

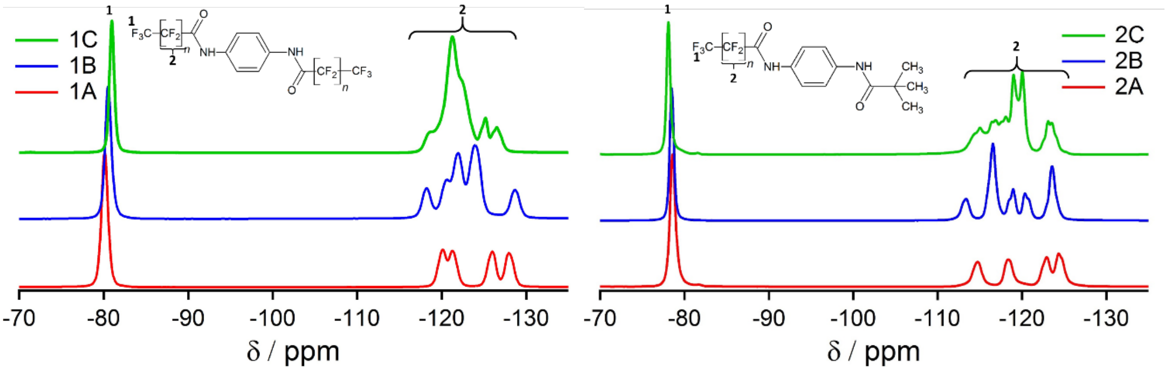

Figure S7. ^{19}F MAS-NMR spectra of series **1** (left) and series **2** (right).

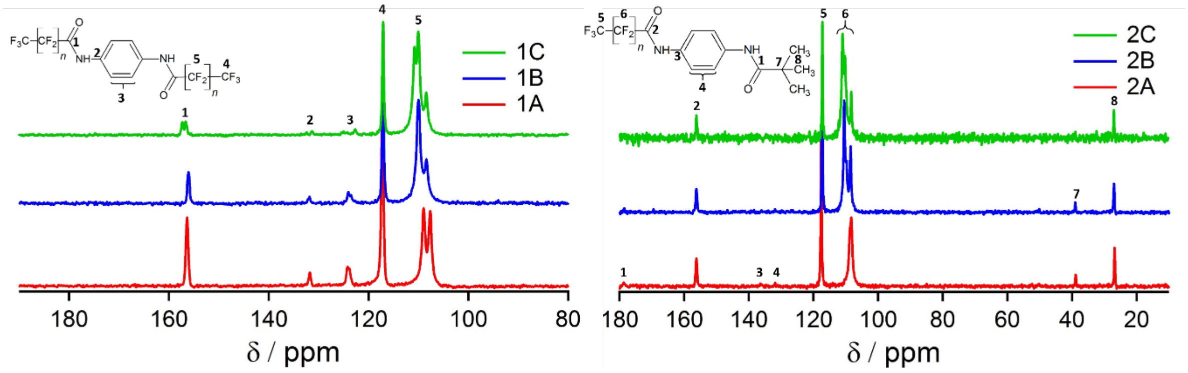

Figure S8. $^{13}C\{^{19}F\}$ CP MAS-NMR spectra of series **1** (left) and series **2** (right).

5: Different coordination of the two series

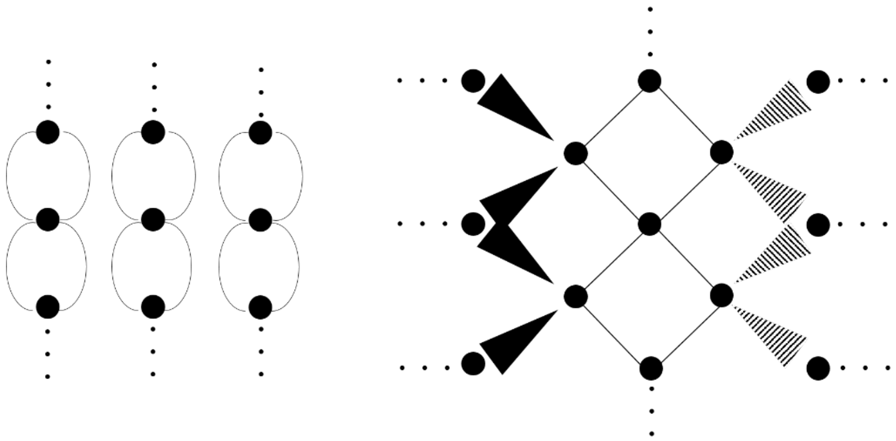

Figure S9. Hydrogen bond pattern of series **1** (left) and series **2** (right) in the so-called Etter notation [2]. The big black dots resemble molecules and the lines resemble hydrogen bonds. The three small dots represent propagation of the pattern in this direction.

6: Different global packing of **2A** compared to **2B** and **2C.**

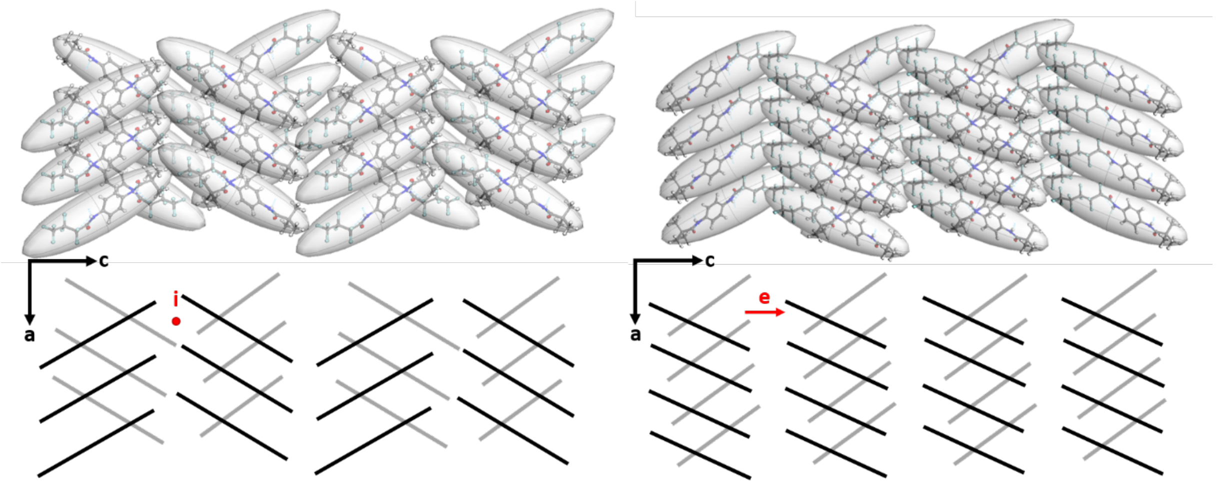

Figure S10. Difference in the global packing of **2A** (left) and series **2B** and **2C** (right). In the sketch on the bottom the black lines resemble the orientation of the top layer and the grey lines resemble the 2nd layer. In red, the symmetry element that determines the space group is depicted.

[2] M. C. Etter, Acc. Chem. Res. 1990, 23, 120

7: Structure models of **2A** in the structure type of series **1**

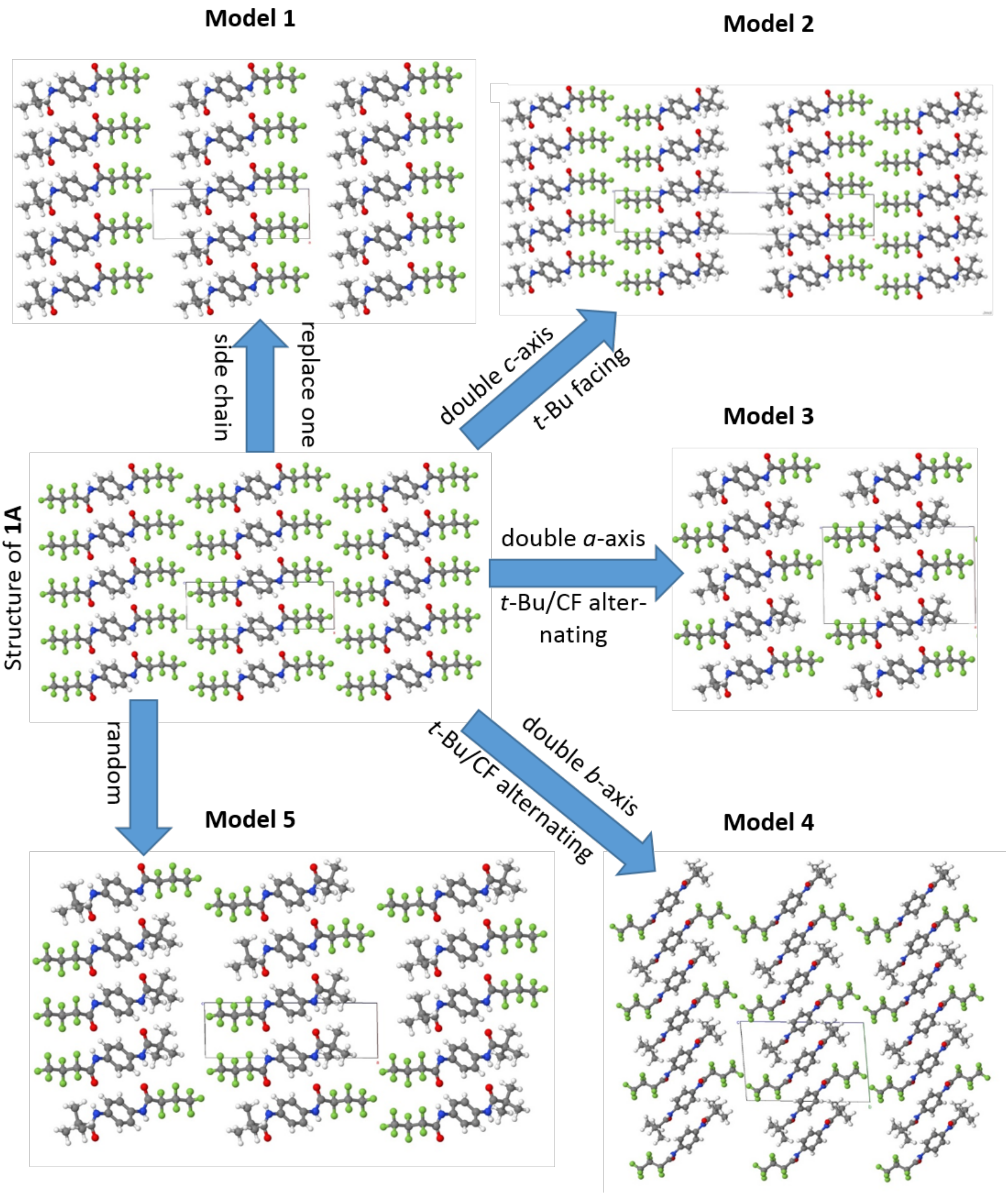

Figure S11. Possibilities of packing **2A** in the structure of **1A.**

To investigate the driving force that leads to the different structure types models of all molecules were built in the structure of the respective molecule of the other series. Since the molecules of series **1** are symmetric, there is only one way to place them into the unit cells of series **2**. For the asymmetric molecules of series **2**, different options arise as depicted for **2A** in Figure S11. These options are:

Model 1: The space symmetry is reduced from $P\overline{1}$ to $P1$ while maintaining the original metric. One side chain of **1A** is then replaced by a *t*-Bu group. The resulting unit cell consists of one molecule leading to a model where *t*-Bu and the CF groups are facing each other at the interlayer gap.

Model 2: The *c*-axis is doubled creating two molecules within the unit cell. The molecules are placed in a way that similar side chains, the t-Bu groups and the fluorinated alkyl chains from neighbouring molecules face each other.

Model 3: The *a*-axis is doubled and the CF chains are replaced by *t*-Bu in a way that they are alternating along the *a*-axis. The unit cell consists of two molecules.

Model 4: The *b*-axis is doubled and the CF chain is replaced by *t*-Bu in a way that they are alternating along the *b*-axis. The unit cell consists of two molecules.

Model 5: Finally, a random combination of all models is possible. Here, however, the unit cell increases dramatically.

For models 1, 2 and 4 the local binding situations around the NH···O hydrogen bonds is similar. In all cases, both the NH and the carbonyl function are terminated by the same side chain. Thus, for the comparison between packing pattern **1** and **2,** both models will lead to the similar results. Models 3 shows a different local environment. Here, the NH and the carbonyl function are terminated by different side chains. Therefore, DFT calculations were restricted to models 1 and 3. They lead to very similar energies per molecules. Doubling one unit cell axis results in an alternating pattern of longer (2.4 Å) and shorter (2.1 Å) hydrogen bond lengths due to the additional degrees of freedom as the molecules are now able to rotate against each other. Model 5 is a superposition of the other models and will show similar results. Thus, for the discussion within the manuscript the smallest model with only one molecule in the asymmetric unit was chosen for the comparison with molecules of series **1** calculated in packing pattern **2**, in order to keep the number of variable parameters similar.

8: Scanning electron microscopy of self-assembled platelets of symmetric 1,4-benzene bisamides **1B**-**C** and asymmetric 1,4-benzene bisamides **2B**-**C**

For the preparation of SEM samples from dispersions, a drop was cast on a clean silicon wafer. The supernatant solvent was removed with a filter paper and the sample was dried at ambient conditions. Subsequently, the sample was sputtered with a platinum layer with a layer thickness of 1.3 nm using a Cressington 208HR sputter coater. SEM measurements were performed with a field emission scanning electron microscope (Zeiss LEO 1530) using an accelerating voltage of 3 kV.

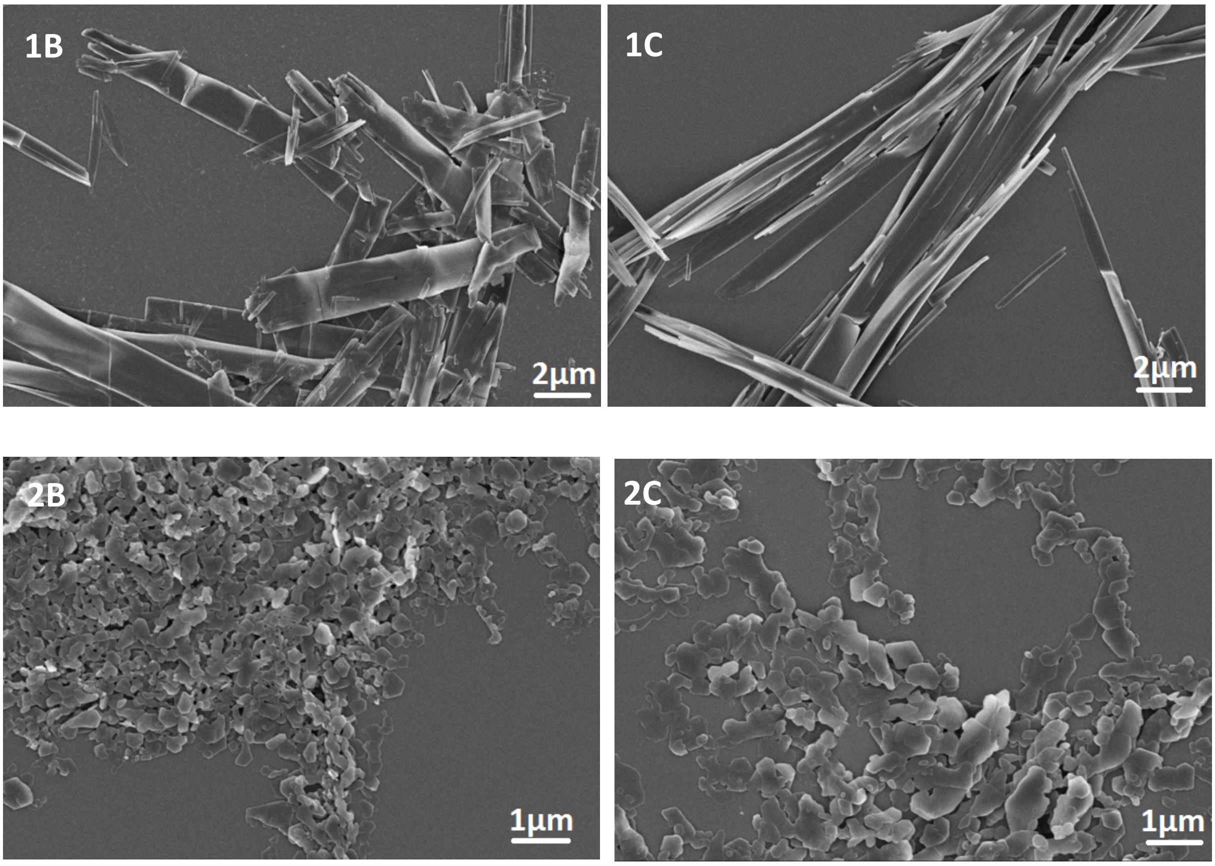

Figure S12. Additional SEM image of platelets of symmetric 1,4-benzene bisamides **1B**, **1C** and asymmetric 1,4-benzene bisamides **2B** and **2C**.

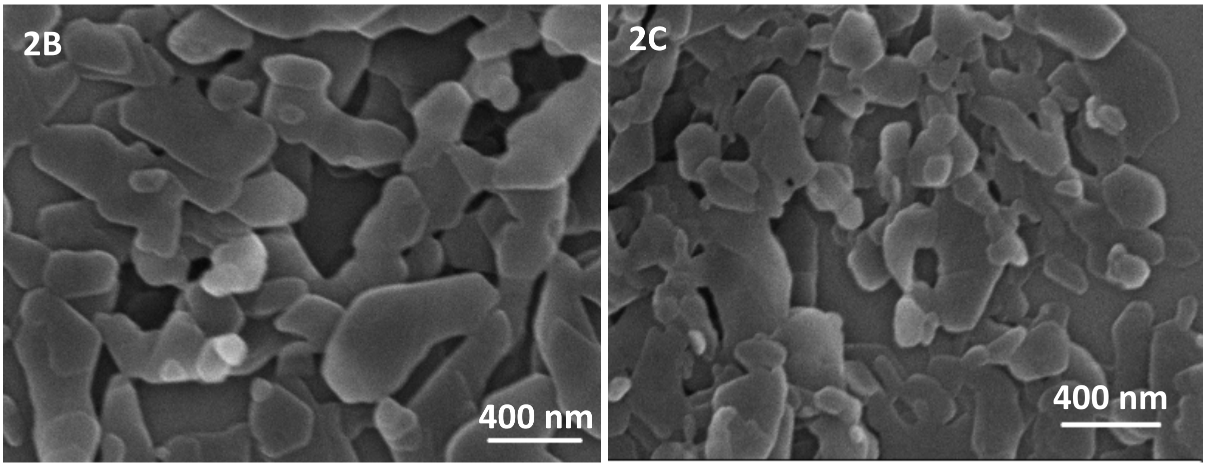

Figure S13. Additional SEM of asymmetric 1,4-benzene bisamides **2B** and **2C** with higher magnification.

9: AFM of self-assembled platelets of both series

AFM measurements were performed using a Veeco dimension 3100 atomic force microscope equipped with a NanoScope IV controller. Bruker OTESPA-R3 silicon cantilevers were used in tapping mode. Square images were captured with 512 lines and 512 points per line and evaluated using Bruker NanoScope Analysis software (version 1.40). Prior to analysis, the images were flattened (1st order). To analyze the layer terraces, small sections from crystals' top surfaces were used. A step analysis, which considers many parallel lines to reduce noise in z direction, gave a profile of the terraces along the x axis. From this profile, tilts were removed using the evaluation software. Parallel lines were fitted to the different terrace levels and the lines' distance was measured to obtain the terrace heights.

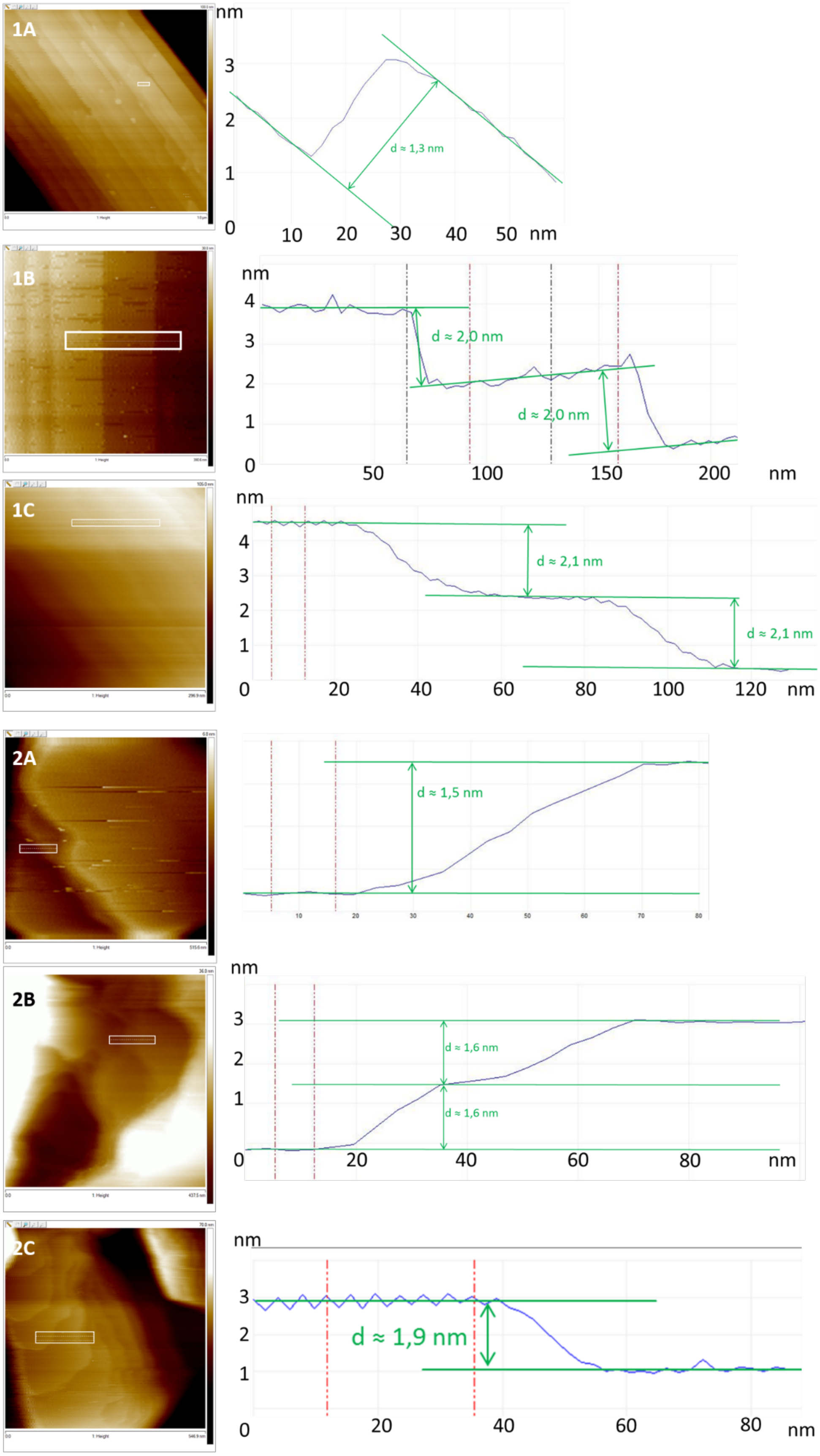

Figure S14. Topographical AFM image (left) of a platelet surface based on **1A**, **1B, 1C**, **2A**, **2B** and **2C** with corresponding height profile (right) of the white rectangle as indicated in the AFM image.

10: Wetting behavior with water on vapor deposited thin films of symmetric 1,4-benzene bisamides **1A** and asymmetric 1,4-benzene bisamides **2A**

As representative model compounds for their wetting behaviour with water by means of contact angle measurements, the symmetric 1,4-benzene bisamides **1A** and asymmetric 1,4-benzene bisamides **2A** were chosen. Both films were prepared by physical vapour deposition.

Subsequently, contact angles on the flat surfaces of the thin films were determined with the sessile drop method using a Krüss DSA25S drop shape analyzer. For each thin film, the average contact angle of at least five measurement was determined (**Figure S14**). The average contact angle for **1A** was found to be 114.9° and for **2A** 104.6°, respectively.

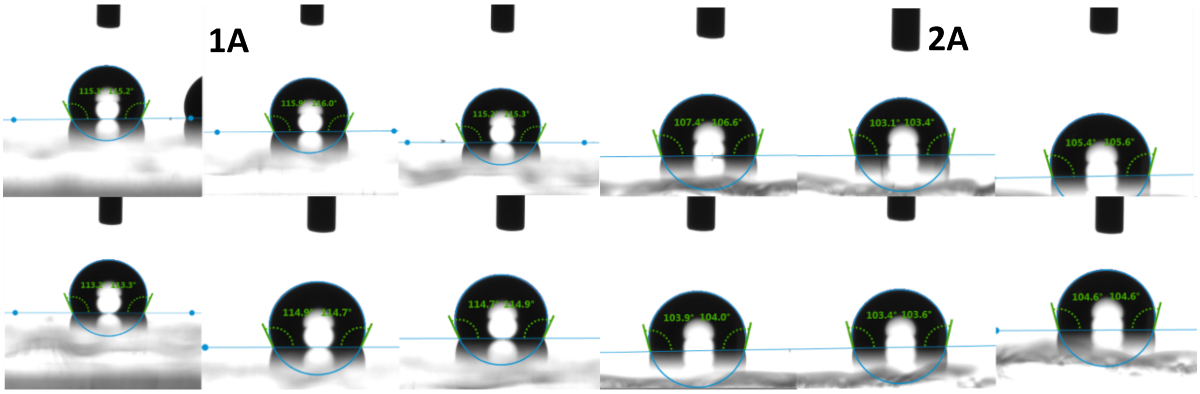

Figure S15. Photographs of contact angle measurements on thin films of **1A** (left) and **2A** (right).
